# Quantum engineering of spin and anisotropy in magnetic molecular junctions

**DOI:** 10.1038/ncomms9536

**Published:** 2015-10-12

**Authors:** Peter Jacobson, Tobias Herden, Matthias Muenks, Gennadii Laskin, Oleg Brovko, Valeri Stepanyuk, Markus Ternes, Klaus Kern

**Affiliations:** 1 Max Planck Institute for Solid State Research, Heisenbergstrasse 1, 70569 Stuttgart, Germany; 2 Max Planck Institute of Microstructure Physics, Weinberg 2, 06120 Halle(Saale), Germany; 3 Institute de Physique de la Matière Condensée, École Polytechnique Fédérale de Lausanne, 1015 Lausanne, Switzerland

## Abstract

Single molecule magnets and single spin centres can be individually addressed when coupled to contacts forming an electrical junction. To control and engineer the magnetism of quantum devices, it is necessary to quantify how the structural and chemical environment of the junction affects the spin centre. Metrics such as coordination number or symmetry provide a simple method to quantify the local environment, but neglect the many-body interactions of an impurity spin coupled to contacts. Here, we utilize a highly corrugated hexagonal boron nitride monolayer to mediate the coupling between a cobalt spin in CoH_*x*_ (*x*=1,2) complexes and the metal contact. While hydrogen controls the total effective spin, the corrugation smoothly tunes the Kondo exchange interaction between the spin and the underlying metal. Using scanning tunnelling microscopy and spectroscopy together with numerical simulations, we quantitatively demonstrate how the Kondo exchange interaction mimics chemical tailoring and changes the magnetic anisotropy.

## Abstract (web summary)

The spins of single molecules and defect centres possess properties which can be strongly influenced by their material contacts in electrical junctions. Here, the authors study the coupling between cobalt hydride complexes and a Rh(111) contact, mediated through a hexagonal boron nitride layer.

## Graphical abstract

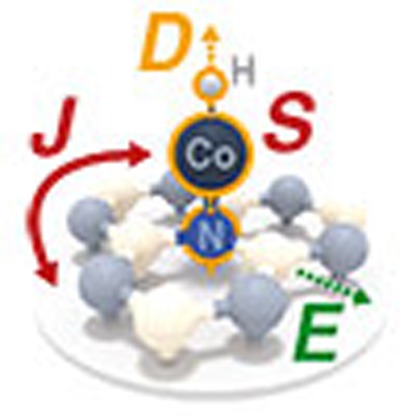

Magnetic anisotropy defines the stability of a spin in a preferred direction^1^,^2^,^3^. For adatoms on surfaces, the low coordination number and changes in hybridization can lead to dramatic enhancement of magnetic anisotropy^2^,^3^,^4^,^5^. Surface adsorption site and the presence of hydrogen has been shown to alter the magnetic anisotropy of adatoms on bare and graphene covered Pt(111)^6^,^7^,^8^. Furthermore, the exchange interaction and strain has been invoked for 3*d* adatoms on Cu_2_N islands where the adatom position on the island affects the observed magnetic anisotropy^9^,^10^. Studies on single molecule magnets (SMMs) containing 3*d* or 4*f* spin centres have revealed that chemical changes to the ligands surrounding the spin affect the magnetic anisotropy^11^,^12^,^13^. However, the most important factor for maintaining magnetic anisotropy in SMMs is a low coordination environment and a high axial symmetry^3^,^14^,^15^.

Magnetic anisotropy is not guaranteed in SMMs or single spin centres on coupling to contacts^9^,^16^. The spin interacts with the electron bath through the exchange interaction leading to a finite state lifetime and the decay of quantum coherence^17^,^18^. Additionally, the scattering of the spin with the electron bath results in an energy renormalization of the spin's eigenstate energy levels, similar to the case of a damped harmonic oscillator^17^. In practice, this leads to a net reduction of the magnetic anisotropy, pushing the system closer to a Kondo state. At the heart of the Kondo effect are spin-flip scattering processes between localized states at the impurity spin and delocalized states in the bulk conduction band, resulting in the formation of a correlated quantum state^19^. The Kondo regime is reached when the magnetic moment of the impurity spin is screened by the electron bath, with the exchange interaction defining the relevant energy scale, the Kondo temperature (*T*_K_). High-spin systems with a total spin *S*>½ have the potential for both magnetic anisotropy and the Kondo effect^20^,^21^. Thus, the Kondo exchange interaction with the electron bath can force the impurity spin into a competing Kondo state, where antiferromagnetic coupling with the reservoir reduces or even quenches the magnetic moment. The outcome of this competition can be determined in local transport measurements, but few quantitative measures of this competition exist.

Here, we study CoH_*x*_ complexes coupled to a spatially varying template, the hexagonal boron nitride (h-BN) moiré on Rh(111), to observe, model and quantify how the environment influences magnetic anisotropy. The h-BN monolayer, a wide bandgap two-dimensional material, decouples and mediates the interactions between CoH_x_ and the underlying Rh metal while lattice mismatch leads to a spatial corrugation resulting in an enlarged unit cell with 3.2 nm periodicity corresponding to 13 BN units on top of 12 Rh atoms^22^,^23^. The local adsorption configuration of CoH_*x*_ on the h-BN is conserved across the moiré unit cell, with the large number of inequivalent adsorption sites allowing us to explore how hybridization affects magnetic anisotropy. To complement our experimental observations, we model transport through the CoH_*x*_ complexes using Hamiltonians that incorporate magnetic anisotropy as well as coupling to the environment. This is accomplished by parameterizing the environment through use of a dimensionless coupling constant −*Jρ*_0_, describing the strength of the Kondo exchange interaction, −*J*, between the localized spin and the electron density *ρ*_0_ of the substrate near the Fermi level.

## Results

### STM topography and local spectroscopy

**Figure 1:**
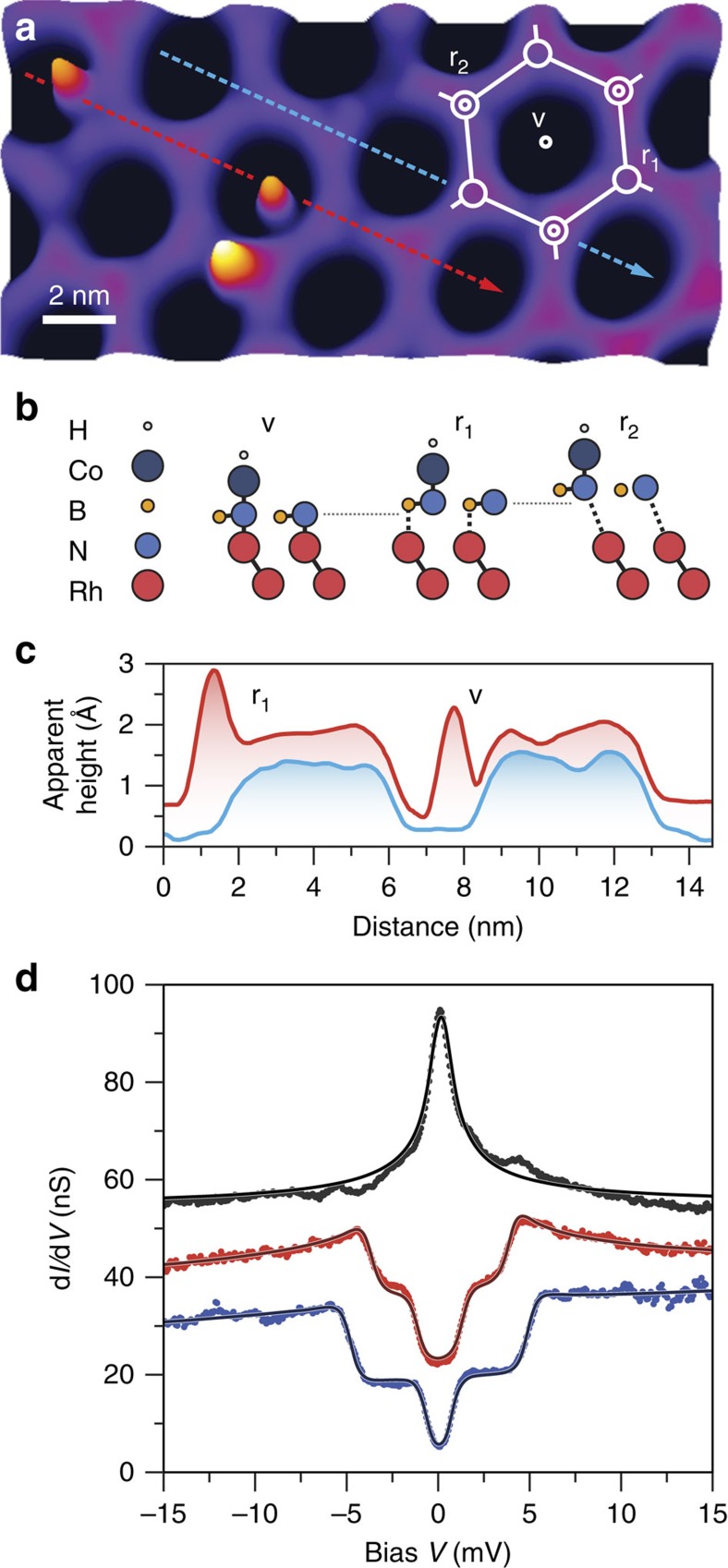
CoH_*x*_ adsorbed on a h-BN/Rh(111) surface. (**a**) Constant current STM topography with three CoH_*x*_ complexes (protrusions) adsorbed on different sites (15 × 7 nm^2^ image size, *V*=−100 mV, *I*=20 pA, *T*=1.4 K). High-symmetry points of the moiré are marked by the white overlay. (**b**) Sketch of the atom positions for the adsorption of CoH. The h-BN registry with Rh(111) shifts across the moiré unit cell with three high-symmetry sites: at the valley site (v) the Rh is directly underneath the N, whereas for the two unequal rim sites (r_1_ and r_2_) changes in the registry and distance to the surface are observed. (**c**) Line profiles along the dashed lines indicated in **a** show two CoH_*x*_ systems with adsorption sites r_1_ and v (red line) and a h-BN reference cut (blue line, offset by 0.5 Å). (**d**) Differential conductance (d*I/*d*V*) curves versus bias voltage of three different CoH_*x*_ systems (Stabilization set point: *I*=500 pA, *V*=−15 mV, *T*=1.4 K, curves vertically offset for clarity). The upper curve (grey) shows a spin-½ Kondo resonance centred at zero bias. The two lower curves (red and blue) show step-like conductance increases symmetric around zero bias indicating a spin-1 system. Solid black lines are least-square fits using a perturbative transport model.

Figure 1a shows a representative scanning tunnelling microscopy (STM) topograph of the h-BN/Rh(111) moiré with isolated CoH_*x*_ (*x*=1,2) complexes, line profiles across the h-BN indicate CoH_*x*_ can adsorb at multiple positions within the moiré (Fig. 1b)^24^. On these CoH_*x*_ complexes we measure the differential conductance (d*I/*d*V*) against the applied bias voltage *V* between tip and sample at low-temperature (*T*=1.4 K) and zero magnetic field (*B*=0 T, details see Methods section). The spectra can be divided into two broad classes: a sharp peak centred at zero bias or two symmetric steps of increasing conductance at well-defined threshold energies (Fig. 1d). The peak at zero bias is consistent with a spin-½ Kondo resonance while the steps correspond to the onset of inelastic excitations from the magnetic ground state to excited states. The observation of two steps hints at a spin-1 system with zero field splitting. The two lower spectra (Fig. 1d; red and blue curves) are measured on CoH at different parts of the moiré and share the same characteristics but the step positions vary.

### Adsorption site and magnetic moments by DFT

**Figure 2:**
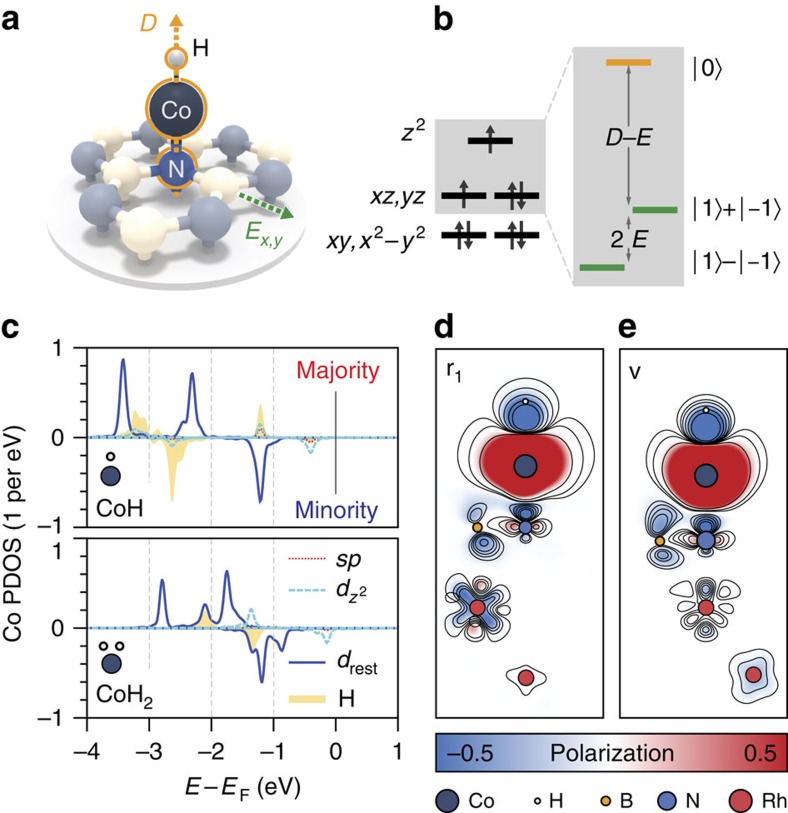
CoH and CoH_2_ density of states. (**a**) Ball and stick model of the adsorption of CoH on h-BN. The linear adsorption geometry of CoH on the N atom is emphasized and marks the main (axial) magnetic anisotropy (*D*) along the *z*-axis. Additional transverse anisotropy (*E*) in the *x*–*y* plane further breaks the symmetry. (**b**) Schematic linear crystal field splitting diagram for the 3*d*^8^ shell of Co highlighting the origin of the axial (*D*) and transverse (*E*) magnetic anisotropy. The magnetic ground state is an antisymmetric superposition of *m*_z_= |+1〉 and |−1〉 states (*m*_z_ is the magnetic moment in units of the reduced Planck constant *ℏ* in *z*-direction), the first excited state is the symmetric superposition, and the second excited state is *m*_z_= |0〉. (**c**) Plots of the majority and minority spin projected density of states (PDOS) for CoH and CoH_2_. The difference in majority and minority spin spectral weights indicate that CoH has a total spin *S*=1 and CoH_2_ has *S*=½. Plot of the asymmetry between majority and minority PDOS for CoH adsorbed on N at the r_1_ (**d**) and v (**e**) high-symmetry points.

We employ density functional theory (DFT) to correlate the magnetic properties of CoH_*x*_ with the local adsorption configuration. Our calculations (see Methods section, Supplementary Fig. 1 and Supplementary Note 1) show that adsorption in the BN hexagon, that is, hollow site, is preferable for bare Co. The addition of hydrogen shifts the preferred adsorption site to N, with the hollow site adsorption energy consistently higher. For CoH complexes the preferred hydrogen position was found to be either exactly on top of Co or tilted towards the nearest B atom (Fig. 2a). An important consequence of the N adsorption site is the linear crystal field acting on the cobalt (that is, N–Co–H) removing the fivefold degeneracy of the *d*-levels (Fig. 2b).

In Fig. 2c the spin-resolved, symmetry decomposed local density of states of CoH and CoH_2_ adsorbed in the h-BN valley are plotted. The atomic *d*-levels are exchange-split roughly 1.2 eV due to the intrinsic Stoner exchange giving a bare Co adatom a magnetic moment of 2.2 Bohr magnetons (*μ*_B_). Formation of CoH leads to hybridization of the H *sp* orbitals and the Co *d*_z^2^_ orbitals, slightly reducing the magnetic moment to 2.0 *μ*_B_, equivalent to a 3*d*^8^ configuration (Fig. 2b). The second hydrogen changes the picture significantly, with the *sp-d* hybridization sufficient to bring the Co *d*-levels closer together, reducing the magnetic moment to 1.2 *μ*_B_ resulting in a 3*d*^9^ configuration. Addition of a third hydrogen results in a complete quenching of the magnetic moment. Therefore, from the combination of our spectroscopic observations and DFT calculations we identify CoH as an effective spin-1 and CoH_2_ as spin-½ system.

Figure 2d,e shows the spin density distribution for CoH in a N adsorption configuration at two high-symmetry points of the moiré. The strong vertical bond between Co and N leads to an effective spin-polarization along this axis and can be expected to provide the system with out-of-plane magnetic anisotropy. The hydrogen is not rigidly pinned to the cobalt and tilting of the hydrogen combined with the underlying lattice mismatch reduces the C_3v_ symmetry and introduces small shifts in the *d*_xz_, *d*_yz_ levels producing a non-negligible in-plane component of the anisotropy.

### Local spectroscopy in magnetic fields

**Figure 3:**
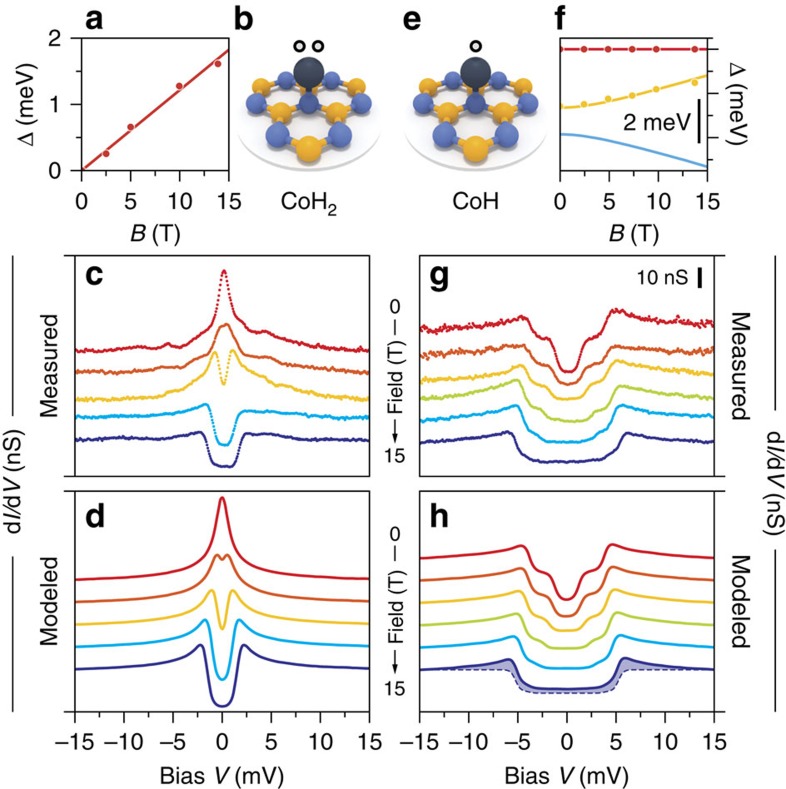
Magnetic field behaviour of CoH_2_ and CoH. (**a**) Zeeman splitting of the spin-½ states of a CoH_2_ complexin magnetic field. Dots mark the energy differences as determined by least-square fits of the perturbation model to the experimental data in (**c**). The regression line corresponds to a gyromagnetic factor *g*=2.0±0.1. (**b**) Sketch of the CoH_2_ complex adsorbed on a N site. (**c**) Evolution of the differential conductance of a CoH_2_ complex in an external magnetic field normal to the surface (*B*=0, 2.5, 5, 10 and 14 T; *T*=1.4 K). (**d**) Simulated spectra using a third order perturbation model and a constant coupling to the substrate of −*Jρ*_0_=0.1 and *g*=2.0. (**e**) Sketch of the spin-1 CoH complex adsorbed on a N site. (**f**) State energy evolution in magnetic field along the out-of-plane anisotropy axis. Dots mark the experimentally determined step positions, full lines are the calculated eigenstate energies of the model Hamiltonian (see text) using magnetic anisotropy parameters of *D*=−4.8 meV, *E*=0.6 meV and *g*=2.2. (**g**) Evolution of the differential conductance of a CoH system in an external magnetic field normal to the surface (*B*=0, 2.5, 5, 7.5, 10 and 14 T; *T*=1.4 K). (**h**) Simulated spectra using the parameter from (**f**) and −*Jρ*_0_=0.1. The 14 T spectrum is shown together with a second order perturbation theory model, that is, −*Jρ*_0_=0 (dashed line), to highlight the necessity of third order contributions. Curves in (**c**,**d**) and (**g**,**h**) are shifted vertically for better visibility.

To model the experimentally observed tunnelling spectra and to determine the magnetic anisotropy we use a phenomenological spin Hamiltonian including the Zeeman energy and magnetic anisotropy:


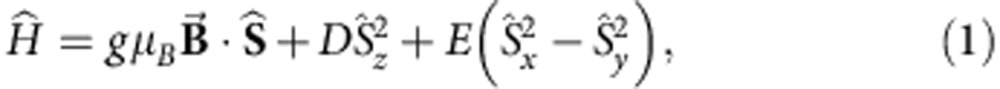


with *g* as the gyromagnetic factor, 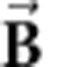 the magnetic field, 
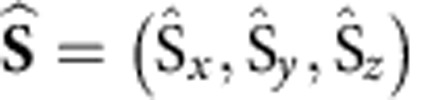
 the total spin operator, and *D* and *E* as the axial and transverse magnetic anisotropy^9^,^10^,^25^,^26^,^27^. Transport through the junction is calculated using a Kondo-like interaction 
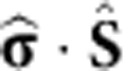
 between the tunnelling electrons and the localized spin system, with 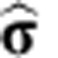 as the standard Pauli matrices. We account for scattering up to third order in the matrix elements by considering additional exchange processes between the localized spin and substrate electrons of the form^28^,^29^ (Supplementary Fig. 2, Supplementary Note 2):


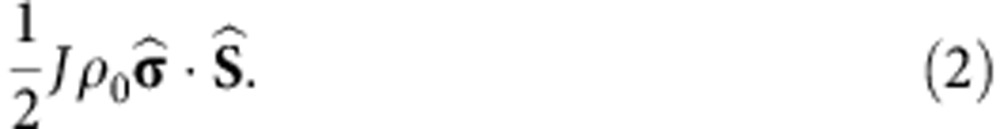


to confirm the magnetic origin of the spectroscopic features in CoH and CoH_2_, we measure the differential conductance at magnetic fields up to 14 T normal to the surface. Figure 3c shows experimental spectra taken over one CoH_2_ complex and Fig. 3d the model calculations for the Kondo resonance. Applying an external magnetic field introduces Zeeman splitting to the spin–½ system (Fig. 3a). At low magnetic fields, 2.5 T, the peak broadens and the differential conductance of the resonance is reduced. Increasing the field to 5 T, a clear splitting of the Kondo resonance is observed. For the highest fields, the degeneracy of the spin-½ state is effectively lifted, resulting in a strong reduction of the Kondo resonance and the appearance of an inelastic excitation gap. We can reproduce the peak and its splitting by our perturbative model (Fig. 3d) even though at high fields the peak-like conductance is weaker in the experimental data than expected from the model calculation. This indicates that the Kondo temperature of the system lies close to the base temperature of our experiment^29^.

Increasing the external magnetic field has two effects on the spin-1 CoH; Zeeman splitting separates the steps and the ratio between inner and outer conductance step height decreases (Fig. 3g). At zero field, the ground and first excited states are a superposition of *m*_z_= |+1〉 and |−1〉 states, applying a magnetic field reduces the spin mixing and leads towards a |+1〉 ground and |−1〉 excited state. This accounts for the reduction of the inner step with increased magnetic field, as the transition between ground and first excited state becomes less probable because it would require a change in *m*_z_ of two. Reverting to a purely second order simulation, large deviations are observed at both steps, evidence that coupling of the spin to the substrate conduction electron bath must be considered (Fig. 3h, dashed line). The experimental data fits excellently when including third order terms, that is, assuming a finite −*Jρ*_0_, an out-of-plane anisotropy axis and *g*=2.2±0.2.

### Correlation between coupling and magnetic anisotropy

**Figure 4:**
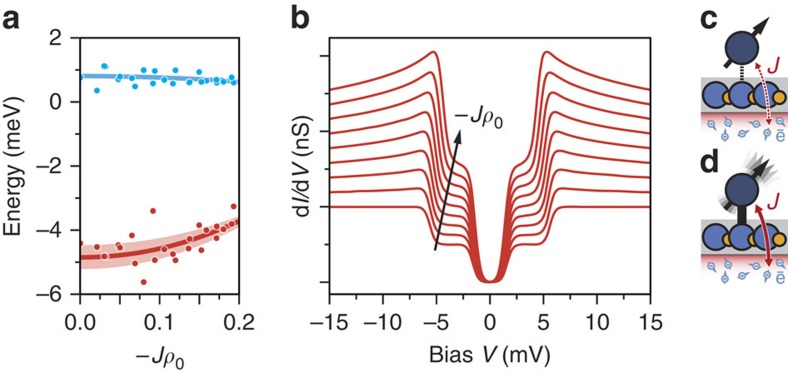
Influence of environmental coupling on CoH spectra. (**a**) Experimentally determined *D* and *E* (red and blue dots) parameters plotted versus the coupling strength−*Jρ*_0_. Full lines show the expected renormalization of *D* and *E* due to virtual coherences calculated with a Bloch-Redfield approach taking exchange scattering with the dissipative substrate electron bath into account. Shaded region shows the experimental uncertainty. (**b**) Computed differential conductance for different coupling strengths between the localized spin and the electrons of the substrate ranging from −*Jρ*_0_=0 to −*Jρ*_0_=0.2. At stronger couplings (−*Jρ*_0_>0) an increase of the outer step's shoulder is expected concomitant with a reduction of the energy position of the outer step. This is equivalent to a reduced anisotropy energy *D*. (**c**,**d**) Schematic diagram showing the effect of exchange. When the exchange coupling, *J*, between the local spin and the conduction electron bath is weak, a large magnetic anisotropy, *D*, is observed (**c**). As exchange coupling to the substrate strengthens, the magnetic anisotropy is reduced driving the system closer to a Kondo state (**d**).

Evaluation of >30 CoH complexes (Fig. 4a) shows no sharp distribution of the anisotropy parameters *D* and *E*. A transition of the main anisotropy axis into the surface plane occurs when 3*E*>|*D*|, therefore we have only considered complexes with a clear out-of-plane anisotropy determined by the criterion (|*D*|)/(3*E*)>1.5; a representative spectrum with in-plane anisotropy is shown in Supplementary Fig. 3. By considering the values of −*Jρ*_0_ from our fits, we observe a correlation between the magnetic anisotropy and coupling with the substrate, −*Jρ*_0_. The red branch in Fig. 4a shows that as the substrate coupling increases, the axial magnetic anisotropy decreases. These results are in line with predictions that increased coupling shifts energy levels. The solid red line shows the best fit to our data and follows the trend *D*=*D*_0_(1−*α*(*Jρ*_0_)^2^), where *α* is a constant describing the bandwidth of the Kondo exchange interaction. The shaded red region accounts for the possible range of *α* by considering an effective bandwidth of *ω*_0_=0.4–1.2 eV (see Supplementary Information). For 0.1<−*Jρ*_0_<0.2 the variation in magnetic anisotropy fits exceptionally well, but for small values of −*Jρ*_0_, some spread in the axial anisotropy is observed. These fluctuations are not accounted for in our model and indicate that for small −*Jρ*_0_ additional factors such as strain or defects may contribute to the magnetic anisotropy. While the axial anisotropy shows clear dispersion, the transverse anisotropy is essentially constant (Fig. 4a, blue).

Figure 4b shows the influence of −*Jρ*_0_ on the tunnelling spectra calculated using a Bloch-Redfield approach to incorporate virtual correlations between the ground and excited states due to the coupling with the dissipative spin bath in the substrate assuming a flat density of states and an effective bandwidth of *ω*_0_=1 eV (Supplementary Fig. 4, Supplementary Note 3)^9^,^17^,^18^. As −*Jρ*_0_ is increased, virtual correlations lead to renormalization and reduce the level splitting. This is observed experimentally as a reduction of the axial magnetic anisotropy. Furthermore, higher order scattering processes in the tunnelling influence the conductance leading to an enhanced shoulder at the outer energy step that changes the contours of the spectrum (Supplementary Fig. 5). The symmetric peaks shift towards zero bias as −*Jρ*_0_ increases indicating that correlations drive the anisotropic spin-1 system closer to the Kondo state. Figure 4c schematically depicts the observed trend, when the spin is weakly coupled to the conduction electrons the magnetic anisotropy is stabilized. Increasing the exchange interaction introduces correlations between the excited spin states and the conduction electrons, leading to a net reduction in the magnetic anisotropy (Fig. 4d).

## Discussion

In conclusion, our results show that the Kondo exchange interaction modulates the magnetic anisotropy of single spin CoH complexes. The role of exchange was quantitatively determined by exploiting the corrugated h-BN moiré structure. In conjunction with third order perturbation theory simulations, we extracted the precise values of the spin coupling to the environment and its influence on the magnetic anisotropy. Kondo exchange must be considered an additional degree of freedom—beyond local symmetry, coordination number and spin state—for spins connected to contacts. This parameter is non-local and therefore expected to be discernable at surfaces, in junctions and perhaps in bulk SMM materials.

## Methods

### Sample preparation

The Rh (111) surface was prepared by multiple cycles of argon ion sputtering and annealing to 1100 K. On the final annealing cycle borazine (B_3_N_3_H_6_) was introduced at a pressure of 1.2 × 10^−6^ mbar for 2 min resulting in a monolayer h-BN film. Cobalt was deposited onto a cold, ∼20 K, h-BN surface via an electron beam evaporator. Hydrogen is the predominant component of the residual gas background and is responsible for the formation of the cobalt hydride complexes^30^,^31^.

### Local spectroscopy

Scanning tunnelling experiments were performed on a home-built STM/AFM in ultra-high vacuum with a base temperature of 1.4 K and magnetic fields up to 14 T. All spectroscopic (d*I/*d*V*) measurements presented were obtained with an external lock-in amplifier and a modulation voltage of 0.2 mV applied to the bias voltage at a frequency of 799 Hz. The tunnelling set point before the feedback loop was disabled was *V*=−15 mV and *I*=500 pA. Given the distinct spectroscopic fingerprints, that is, steps or peaks, we used spectroscopy to sort our data as either spin-1 or spin-½. In conjunction with the DFT calculations, we assign the spin-½ species as CoH_2_ and spin-1 species as CoH. For measurements on the same adatoms in different external magnetic fields the tip was retracted while the field was ramped and allowed to settle for maximum stability. Supplementary Fig. 6 shows a bare Co adatom spectrum. Supplementary Fig. 7 shows an error analysis. Supplementary Fig. 8 shows a set point dependence.

### Density functional calculations

First principles calculations have been carried out in the framework of the DFT as implemented in the Vienna Ab-initio Simulation Package (VASP) code^32^,^33^. We use the projector augmented-wave technique^34^ where the exchange and correlation were treated with the gradient-corrected PBE functional as formalized by Perdew, Burke and Ernzerhof^35^. Hubbard *U* and *J* values were taken from self-consistent calculations and fitting to experiments to be *U−J*=3 eV (refs 36, 37, 38). Full details are presented as Supplementary Note 1, Supplementary Fig. 1 and Supplementary Tables 1 and 2.

## Additional information

**How to cite this article:** Jacobson, P. *et al*. Quantum engineering of spin and anisotropy in magnetic molecular junctions. *Nat. Commun.* 6:8536 doi: 10.1038/ncomms9536 (2015).

## Supplementary Material

Supplementary Information Supplementary Figures 1-8, Supplementary Tables 1-2, Supplementary Notes 1-3 and Supplementary References
