# Supplementary material for: Quantum engineering of spin and anisotropy in magnetic molecular junctions
Source: Nat Commun. 2015 Oct 12;6:8536. doi: 10.1038/ncomms9536 (PMC4633813; doi:10.1038/ncomms9536)
Supplement: Supplementary Information — Supplementary Figures 1-8, Supplementary Tables 1-2, Supplementary Notes 1-3 and Supplementary References [file ncomms9536-s1.pdf]

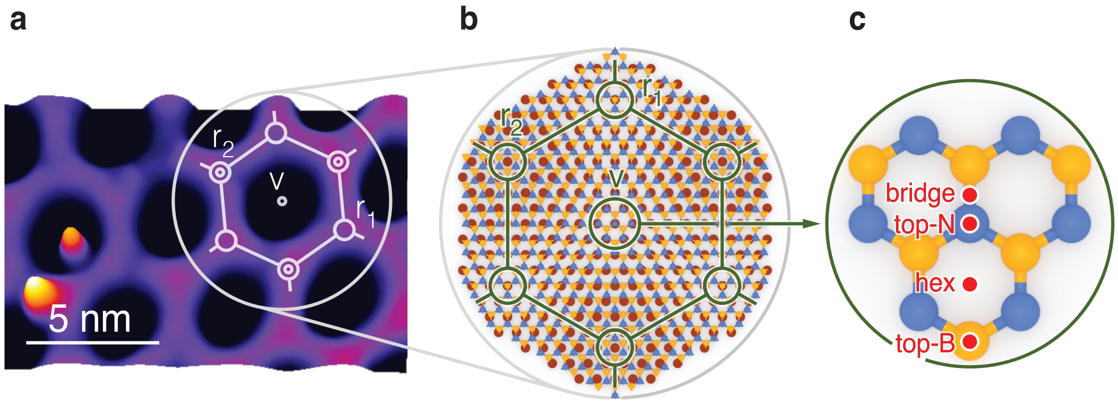

Supplementary Figure 1. **Simulation details for the DFT calculation** (a) A representative constant current STM topography of a portion of the h-BN mesh on Rh(111) (15×7 nm^2 image size, $V = -100$ mV, $I = 20$ pA, $T = 1.4$ K). (b) The three high symmetry sites of the mesh: v (valley) where Rh is directly underneath the N and $r1$, $r2$ (rim) sites where Rh is underneath B and the hollow hexagon site respectively. (c) Considered CoH_x adsorption positions on the BN mesh regardless of the underlying Rh raster: in the hexagonal hollow site (hex), on top of N and B (top-N, top-B) and in the bridge position (bridge).

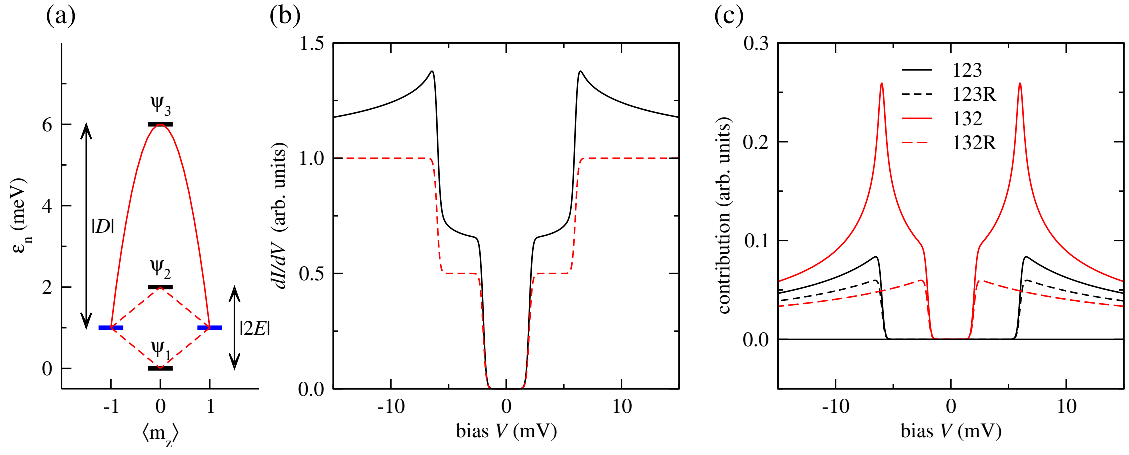

Supplementary Figure 2. **Simulated tunneling spectra of a spin $S = 1$ system (a)** The anisotropy lifts all degeneracies ($D = -5$ meV, $E = 1$ meV) at zero field. The plot shows the state energy over the m_z expectation value $\langle m_z \rangle = \mathrm{tr}\left(\hat{S}_z \left|\Psi_i\right\rangle \left\langle\Psi_i\right|\right)$. **(b)** The total spectrum with $J\rho_0 = 0$ (dashed red line) and -0.1 (full black line). **(c)** The significant higher order processes where the digits refer to the eigenstates of the process ($T = 1$ K, $B = 0$ T).

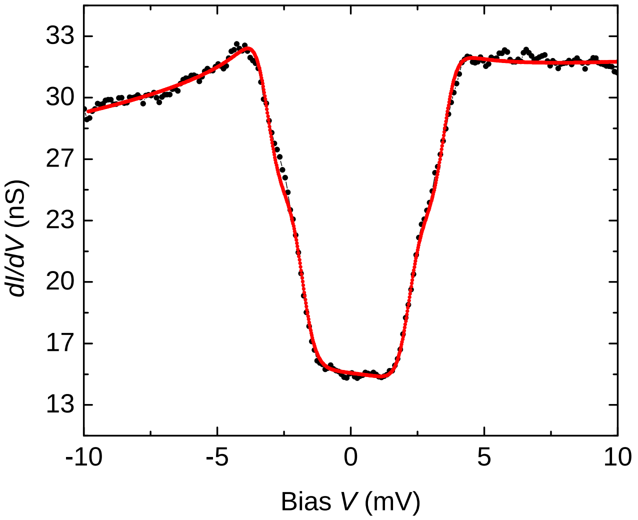

Supplementary Figure 3. **Cobalt hydrogen complexes with in-plane anisotropy:** In rare occasions (few percent of the observations) we find CoH complexes which have a magnetic anisotropy axis in the surface plane of the h-BN. These species reveal themselves by two symmetric steps at similar energy in the differential conductance. A fit to the example spectrum shown here yields: $D = 2.67$ mV, $E = 0.68$ mV, and coupling strength of $J\rho_0 = -0.07$. To simplify the analysis of the main paper such data was neglected.

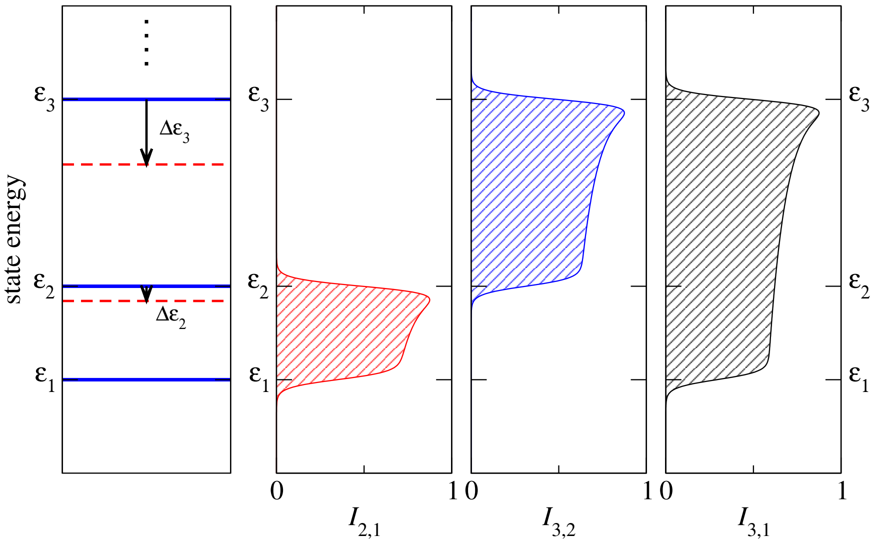

Supplementary Figure 4. **Principle scheme of the shift of the state energies:** The integral contributions $I_{i,j}$ to the energy shift $\Delta\varepsilon_i$ are displayed revealing that for the low lying state at the energy ε_2 only $I_{2,1}$ has weight in the supplementary equation S9, while for ε_3 the weights $I_{3,2}$ and $I_{3,1}$ have to be accounted for.

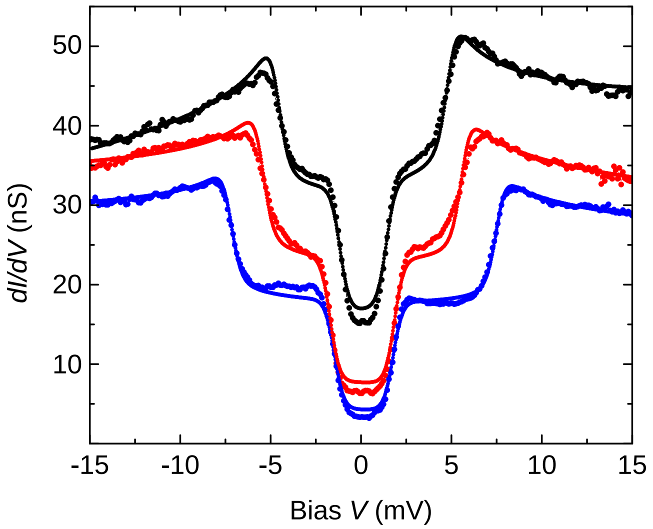

Supplementary Figure 5. **Example spectra for different coupling:** In this figure three spectra with high, middle, and low values of $J\rho_0$ are shown to emphasize the quality of the fits over the entire range. From top to bottom the curves have values of $J\rho_0$: -0.27 ($D = -4.13$ meV), -0.15 ($D = -4.66$ meV), -0.11 ($D = -6.58$ meV). The top two curves have been offset for clarity by 5 and 10 nS, respectively.

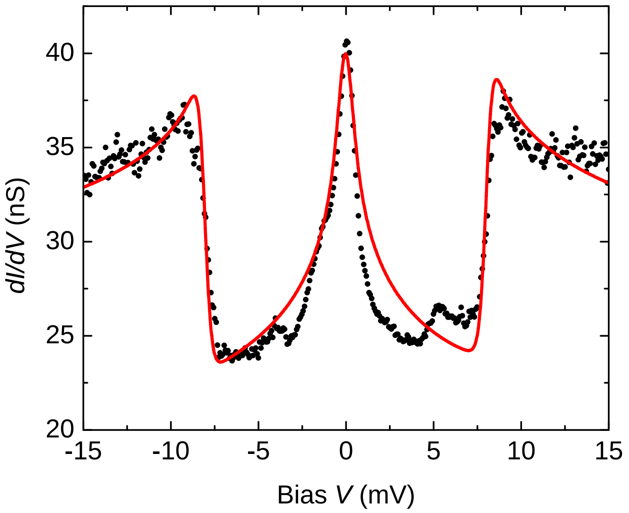

Supplementary Figure 6. **Bare Cobalt with S=3/2:** An example of a bare Co atom on h-BN/Rh(111) that show an effective spin-3/2. The spin 3/2 systems exhibit both the Kondo effect and magnetic anisotropy[1–3]. Fitting this spin 3/2 spectrum in the same manner as the data presented in the main paper reveals an axial anisotropy $D = 3.76$ meV and a transverse anisotropy $E = 0.82$ meV as well as a coupling to the substrate of $J\rho_0 = -0.19$. While the Kondo correlations hinder the exact determination of the coupling to the substrate[2,4], we neglected these systems from our analysis.

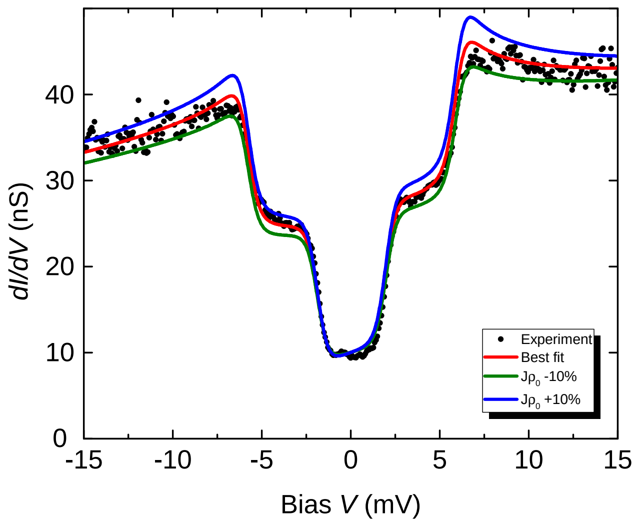

Supplementary Figure 7. **Error estimation of the fit:** A spin-1 CoH with the best $J\rho_0$ fit and two fits with $J\rho_0 \pm 10\%$ of the best-fit value. To help quantify the error, we compute the mean square error of the best fit and imperfect fits. The best fit (red) has a mean square error value of 2.8 %, the fits with ± 10 % $J\rho_0$ have a mean square error of 5.2 % (green) and 11.6 % (blue).

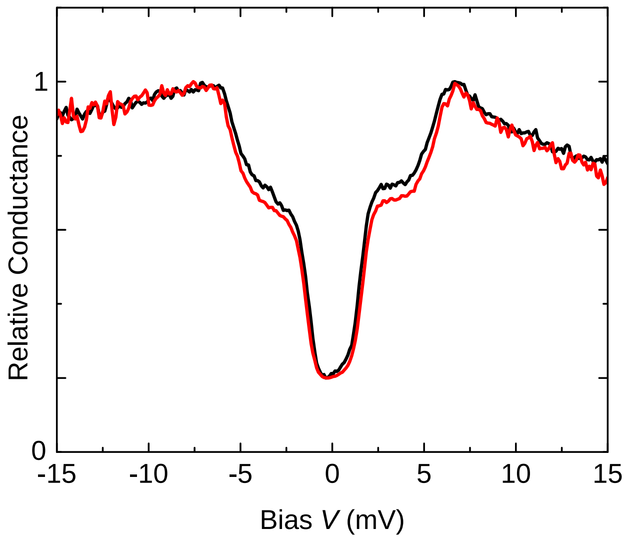

Supplementary Figure 8. **Example spectra for different tunneling setpoint:** Two spectra of a spin-1 CoH complex where the STM current setpoint was changed from 30 MΩ to 2.5 MΩ corresponding to a change in tip sample distance of ≈ 100 pm. While the overall shape is identical, small changes are due to additional hybridization with the tip and non-equilibrium effects[4,5]. Note, however, that all spectra presented in the main manuscript were taken at an identical tunneling setpoint of 30 MΩ.

		hollow	top-B	top-N	bridge
Co	pore	0	5	23	21
Co	r2	0	183	427	348
Co	r1	0	207	351	232
CoH	pore	91	739	0	96
CoH	r2	73	261	0	137
CoH	r1	0	173	n/c	52
CoH_2	pore	140	315	0	34
CoH_2	r2	221	510	0	32
CoH_2	r1	57	599	0	24

Supplementary Table 1. Relative adsorption energies of cobalt hydride complexes in meV relative to the lowest adsorption energy value for the given pore location and hydrogenation degree. "n/c" means that no agreeable electronic convergence could be achieved.

		hollow	top-B	top-N	bridge
Co	pore	2.1	2.2	2.2	2.2
Co	r2	2.1	2.1	2.1	2.2
Co	r1	2.0	2.1	2.2	2.1
CoH	pore	2.0	1.9	2.0	2.0
CoH	r2	2.1	2.1	2.1	2.1
CoH	r1	2.1	2.1	n/c	2.1
CoH_2	pore	1.2	1.6	1.2	1.3
CoH_2	r2	1.3	1.5	1.5	1.6
CoH_2	r1	1.6	1.2	1.5	1.6

Supplementary Table 2. Magnetic moments of cobalt hydride complexes in μ_B. "n/c" stands for no agreeable electronic convergence achieved.

Supplementary Note 1. DETAILS ABOUT THE DENSITY-FUNCTIONAL CALCULATION

First principles calculations have been carried out in the framework of the density functional theory (DFT) as implemented in the VASP code[6,7]. Utilized were the projector augmented-wave technique[8]. The exchange and correlation were treated with the gradient-corrected PBE functional as formalized by Perdew, Burke and Ernzerhof in 1996.[9] We test the robustness of the results by Ceperley-Alder exchange and correlation functionals (LDA)[10] as parametrized in the Perdew and Zunger[11] and Perdew and Wang[12] formalization of the gradient-corrected functional (GGA-PW91).

To account for the on-site Coulomb interaction of the d-levels of Co the LDA+U correction was included in the calculations[13–15] in the spherically invariant form suggested in Ref. 15. Hubbard U and J values were taken from self-consistent calculations to be $U - J = 3$ eV.[16–19] In general the influence of U was found, as expected, to enhance local magnetism, however not changing the validity of any of the conclusions discussed in the present work (especially the attribution of CoH_x complexes to either $S = 1$ or $S = 1/2$).

In terms of geometry most structural parameters were obtained self-consistently by minimizing the interatomic forces to less than 1 meV/Å. First, the equilibrium lattice constant of Rh bulk was established, then the surface was modeled by 10 layers of Rh(111) where the top three layers were allowed to relax vertically. The adsorption of BN on Rh in the valley region was modeled by adjusting the lattice constant of the BN sheet to the Rh lattice parameter and allowing the commensurate BN sheet to fully relax on the Rh(111) surface. For the adsorption configuration at the rim the adsorption heights were increased in accordance to the results of molecular dynamics and ab-initio simulations.[20–24]

For the simulation of CoH_x adsorption a 4×4 in-plane unit cell was used with the BN sheet still kept commensurate to the underlying Rh to preserve the periodicity of the cell. The Co, CoH, and CoH_2 complexes were placed on top of the chosen adsorption sites: hex, top-N, top-B, bridge, see supplementary figure 1. Co was allowed to relax vertically and H atoms were allowed an unconstrained relaxation.

To check the adsorption energy landscape for CoH_x complexes within one BN cell adsorption energy maps were also calculated and were found to be smooth and devoid of local minima, making the choice of discussed adsorption sites a representative one, see supplemen-

tary table . In addition, we have computed the magnetic moments of the cobalt complexes in these adsorption configurations, see supplementary table 2.

Supplementary Note 2. TRANSPORT CALCULATION FOR THE $S = 1$ SPIN

We describe the spin system with a phenomenological Hamiltonian, which is sufficient to fully explain the spectroscopic features observed in our scanning tunneling spectroscopy measurements:

$$\hat{H} = g\mu_B \vec{\boldsymbol{B}} \cdot \hat{\mathbf{S}} + D\hat{S}_z^2 + E\left(\hat{S}_x^2 - \hat{S}_y^2\right). \tag{S1}$$

In this equation g is the gyromagnetic factor, μ_B Bohr's magneton, D determines the axial anisotropy, and E the transverse anisotropy. $\vec{\boldsymbol{B}}$ is the external applied magnetic field and $\hat{\mathbf{S}} = (\hat{S}_x, \hat{S}_y, \hat{S}_z)^T$ the total spin operator with the components ($\hbar = 1$):

$$\hat{S}_x = \begin{pmatrix} 0 & \frac{1}{\sqrt{2}} & 0 \\ \frac{1}{\sqrt{2}} & 0 & \frac{1}{\sqrt{2}} \\ 0 & \frac{1}{\sqrt{2}} & 0 \end{pmatrix}, \quad \hat{S}_y = \begin{pmatrix} 0 & \frac{-i}{\sqrt{2}} & 0 \\ \frac{i}{\sqrt{2}} & 0 & \frac{-i}{\sqrt{2}} \\ 0 & \frac{i}{\sqrt{2}} & 0 \end{pmatrix}, \quad \hat{S}_z = \begin{pmatrix} 1 & 0 & 0 \\ 0 & 0 & 0 \\ 0 & 0 & -1 \end{pmatrix}. \tag{S2}$$

In the absence of a magnetic field the three eigenvectors $|\Psi\rangle_i$ and eigenenergies ϵ_i of supplementary equation S1 are calculated in the m_z basis to

$$\begin{aligned} \varepsilon_1 &= 0, & |\Psi_1\rangle &= -\frac{1}{\sqrt{2}}|+1\rangle + \frac{1}{\sqrt{2}}|-1\rangle, \\ \varepsilon_2 &= 2E, & |\Psi_2\rangle &= +\frac{1}{\sqrt{2}}|+1\rangle + \frac{1}{\sqrt{2}}|-1\rangle, \\ \varepsilon_3 &= E - D, & |\Psi_3\rangle &= |0\rangle, \end{aligned}$$

as shown in supplementary figure 2a for hard axis anisotropy ($D < 0$) and non negligible transverse anisotropy ($E \neq 0$).

To calculate the tunneling spectrum we use a model based on the perturbative approach established by Appelbaum, Anderson, and Kondo[25–28] in which spin-flip scattering processes up to the 2nd order Born approximation are accounted for and which has been previously successfully used on quantum spin systems[4,29]. In this model the transition probability $W_{i \to f}$ for an electron to tunnel between tip and sample and concomitantly changing the spin state

of the CoH complex from its initial (i) to its final (f) state is

$$W_{i\to f} \propto \left(|M_{i\to f}|^2 + \rho_0 J \sum_m \left(\frac{M_{i\to m} M_{m\to f} M_{f\to i}}{\varepsilon_i - \varepsilon_m} + \text{c. c.} \right) \right) \delta(\varepsilon_i - \varepsilon_f). \tag{S3}$$

Here, $M_{i\to j}$ are the matrix elements given by the Kondo-like interaction of the scattering electron $|\varphi\rangle$ with the localized spin of the CoH complex $|\Psi\rangle$

$$M_{i\to f} = \sum_{i',f'} \langle \varphi_{f'}, \Psi_f | \frac{1}{2} \hat{\boldsymbol{\sigma}} \cdot \hat{\mathbf{S}} | \varphi_{i'}, \Psi_i \rangle. \tag{S4}$$

In this equation $|\varphi_i, \Psi_i\rangle$ is the combined state vector of the localized spin and the interaction electron and $\hat{\boldsymbol{\sigma}} = (\hat{\sigma}_x, \hat{\sigma}_y, \hat{\sigma}_z)^T$ is the total spin operator for the spin-1/2 electrons, with $\hat{\sigma}_{x,y,z}$ as the standard Pauli matrices.

The first term in the supplementary equation S3 is responsible for the conductance steps observed in our spectra. While we assume zero field ($B = 0$) and no spin-polarization in the two electron reservoirs of tip and sample, the matrix elements are easily calculated to $|M_{i\to j}|^2 = 0.5$ for $i \neq j$ and $|M_{i\to i}|^2 = 0$ otherwise. This leads at low temperature, i. e. $k_B T \ll \varepsilon_2$, when only the ground state $|\Psi_1\rangle$ is occupied, to two increasing steps in the differential conductance dI/dV with identical amplitude at the energies $\pm\varepsilon_2$ and $\pm\varepsilon_3$ (dashed line in supplementary figure 2(b)):

$$\sigma_1(eV) = \frac{1}{2}\sigma_0 \left[\Theta\left(\tfrac{\varepsilon_2 + eV}{k_B T}\right) + \Theta\left(\tfrac{\varepsilon_2 - eV}{k_B T}\right) + \Theta\left(\tfrac{\varepsilon_3 + eV}{k_B T}\right) + \Theta\left(\tfrac{\varepsilon_3 - eV}{k_B T}\right) \right], \tag{S5}$$

with $\Theta(\epsilon) = [1 + (\epsilon - 1)\exp(\epsilon)]\,[1 - \exp(\epsilon)]^{-2}$ as the thermally broadened step function[30], and σ_0 as the total conductance in the limit of high bias.

The second term of the supplementary equation S3 is due to the 2nd order Born approximation and accounts for scattering processes involving the intermediate state $|\Psi_m\rangle$. At the bias voltage where this process changes from being virtual to real, the denominator approaches zero which leads to a temperature broadened logarithmic divergence in the spectrum:

$$g(\epsilon) = -\int_{-\infty}^{+\infty} d\epsilon'' \int_{-\omega_0}^{+\omega_0} d\epsilon' \frac{1 - f(\epsilon', T)}{\epsilon' - \epsilon''} f'(\epsilon'' - \epsilon, T), \tag{S6}$$

with $f(\epsilon, T) = [1 + \exp(\epsilon/k_B T)]^{-1}$ as the Fermi-Dirac distribution and $f'(\epsilon, T) = \partial f / \partial \epsilon =$

$(k_B T)^{-1}\mathrm{sech}^2[\epsilon/(2k_B T)]$ as its derivation[4,31]. For the tunneling spectra the correct value of the cut-off energy ω_0 is uncritical, but is of crucial important for the energy renormalization, as we will see in the supplementary note 3. The dimensionless scaling factor $-J\rho_0$ accounts for the fact that either the scattering into the intermediate or the final state is performed with electrons originating and ending in the substrate. Here, J is the coupling strength between substrate electrons and the localized spin and ρ_0 the substrate electron density at E_F.

In the case discussed here, with $S = 1$ and all degeneracies broken, the real parts of the matrix elements at zero field are calculated to $\Re(M_{i\to m}M_{m\to f}M_{f\to i}) = -1/4$ for the processes which go over all states and are otherwise zero. Assuming that solely the ground state is thermally populated only the processes $1 \to 2 \to 3$ and $1 \to 3 \to 2$ account to the conductance leading to an additional conductance of:

$$\begin{aligned}\sigma_2(eV) = -\frac{1}{4}\sigma_0 J\rho_0 \ \Big\{&[g(\varepsilon_2+eV)+g(\varepsilon_2-eV)]\left[\Theta\left(\tfrac{\varepsilon_3+eV}{k_BT}\right)+\Theta\left(\tfrac{\varepsilon_3-eV}{k_BT}\right)\right]\\ &+[g(\varepsilon_3+eV)+g(\varepsilon_3-eV)]\left[\Theta\left(\tfrac{\varepsilon_2+eV}{k_BT}\right)+\Theta\left(\tfrac{\varepsilon_2-eV}{k_BT}\right)\right]\Big\}. \qquad \text{(S7)}\end{aligned}$$

The conductance σ_2 changes in a very particular fashion the observed spectra which is the sum of σ_1 and σ_2: Additional peak-like structures arise at the energy ϵ_3 which allow us to determine $J\rho_0$ very precisely from fits of the supplementary equations S5 and S7 to the spectra measured at zero field.

Supplementary Note 3. RENORMALIZATION OF THE EIGENSTATE ENERGIES

Treating the quantum mechanical system $\hat{H}$ (supplementary equation S1) not as a separated system but coupled to the dissipative bath of the substrate electrons we employ a Bloch-Redfield approach to account for the decay of excited states and coherences in the density matrix[32]. Interestingly, this approach leads for the off-diagonal elements of the reduced density matrix of $\hat{H}$ not only to a fast decoherence but additionally to an energy shift of the eigenstates due to the interaction between $\hat{H}$ and the reservoir. We will restrict ourselves to the Kondo-like scattering between the substrate electrons and the localized spin, as

described by supplementary equation S4, up to second order leading to a correction term[2,32]:

$$\Delta\varepsilon_\alpha = (J\rho_0)^2 \sum_n \sum_{n',\alpha'} \frac{\left|\langle\varphi_{n'}, \Psi_n| \frac{1}{2}\hat{\boldsymbol{\sigma}} \cdot \hat{\mathbf{S}} |\varphi_{\alpha'}, \Psi_\alpha\rangle\right|^2}{\varepsilon_\alpha - \varepsilon_n + \epsilon_{\alpha'} - \epsilon_{n'}}. \tag{S8}$$

Knowing the scattering matrix elements and making use of supplementary equation S6 we can rewrite the energy shift to:

$$\Delta\varepsilon_\alpha = \frac{(J\rho_0)^2}{2} \sum_n \int_{-\infty}^{+\infty} d\epsilon \; g(\varepsilon_\alpha - \varepsilon_n + \epsilon) f(\varepsilon_n - \epsilon)(1 - f(\varepsilon_\alpha - \epsilon)). \tag{S9}$$

Supplementary figure 4 illustrates the effect of energy renormalization. The energetically higher excited state at ε_3 is more affected as the low lying state at ε_2.

For the magnetic anisotropy parameters D and E of the CoH system the shift can be approximated to:

$$D(J\rho_s) \approx D_0 \left(1 - \alpha(J\rho_0)^2\right), \quad \text{and} \quad E(J\rho_s) \approx E_0 \left(1 - \beta(J\rho_0)^2\right), \tag{S10}$$

with the coefficients α and β given by the integrals of supplementary equation S9.

SUPPLEMENTARY REFERENCES

[1] Otte, A. F. *et al.* The role of magnetic anisotropy in the Kondo effect. *Nature Physics* **4**, 847–850 (2008).

[2] Oberg, J. C. *et al.* Control of single-spin magnetic anisotropy by exchange coupling. *Nature Nanotechnology* **9**, 64–68 (2013).

[3] von Bergmann, K., Ternes, M., Loth, S., Lutz, C. P. & Heinrich, A. J. Spin-polarization of the split Kondo state. *Phys. Rev. Lett.* **114**, 076601 (2015).

[4] Ternes, M. Spin excitations and correlations in scanning tunneling spectroscopy. *New J. Phys.* **17**, 063016 (2015).

[5] Loth, S. *et al.* Controlling the state of quantum spins with electric currents. *Nature Physics* **6**, 340–344 (2010).

[6] Kresse, G. & Hafner, J. Ab initio molecular dynamics for liquid metals. *Phys. Rev. B* **47**, 558–561 (1993).

[7] Kresse, G. & Furthmüller, J. Efficient iterative schemes for ab initio total-energy calculations using a plane-wave basis set. *Phys. Rev. B* **54**, 11169–11186 (1996).

[8] Blöchl, P. E. Projector augmented-wave method. *Phys. Rev. B* **50**, 17953–17979 (1994).

[9] Perdew, J. P., Burke, K. & Ernzerhof, M. Generalized gradient approximation made simple. *Phys. Rev. Lett.* **77**, 3865–3868 (1996).

[10] Ceperley, D. M. & Alder, B. J. Ground state of the electron gas by a stochastic method. **45**, 566–569 (1980).

[11] Perdew, J. P. & Zunger, A. Self-interaction correction to density-functional approximations for many-electron systems. *Phys. Rev. B* **23**, 5048–5079 (1981).

[12] Perdew, J. P. & Wang, Y. Accurate and simple analytic representation of the electron-gas correlation energy. *Phys. Rev. B* **45**, 13244–13249 (1992).

[13] Anisimov, V. I., Zaanen, J. & Anderson, O. K. Band theory and Mott insulators: Hubbard U instead of Stoner I. *Phys. Rev. B* **44**, 943–954 (1991).

[14] Liechtenstein, A. I., Anisimov, V. I. & Zaanen, J. Density-functional theory and strong interactions: Orbital ordering in Mott-Hubbard insulators. *Phys. Rev. B* **52**, R5467–R5470 (1995).

[15] Dudarev, S. L., Botton, G. A., Savrasov, S. Y., Humphreys, C. J. & Sutton, A. P. Electron-energy-loss spectra and the structural stability of nickel oxide: An LSDA+U study. *Phys. Rev. B* **57**, 1505–1509 (1998).

[16] Steiner, M. M., Albers, R. C. & Sham, L. J. Quasiparticle properties of Fe, Co, and Ni. *Phys. Rev. B* **45**, 13272–13284 (1992).

[17] Osterwalder, J. Correlation effects and magnetism in 3*d* transition metals. *J. Electron Spec. Rel. Phen.* **117–118**, 71–88 (2001).

[18] Gorelov, E., Wehling, T. O., Rubtsov, A. N., Katsnelson, M. I. & Lichtenstein, A. I. Relevance of the complete Coulomb interaction matrix for the Kondo problem: Co impurities in Cu hosts. *Phys. Rev. B* **80**, 155132 (2009).

[19] Wehling, T. O., Balatsky, A. V., Katsnelson, M. I., Lichtenstein, A. I. & Rosch, A. Orbitally controlled Kondo effect of Co adatoms on graphene. *Phys. Rev. B* **81**, 115427 (2010).

[20] Laskowski, R. & Blaha, P. Ab initio study of *h*-BN nanomeshes on Ru(001), Rh(111), and Pt(111). *Phys. Rev. B* **81**, 075418 (2010).

[21] Laskowski, R., Blaha, P. & Schwarz, K. Bonding of hexagonal BN to transition metal surfaces: An ab initio density-functional theory study. *Phys. Rev. B* **78**, 045409 (2008).

[22] Laskowski, R., Blaha, P., Gallauner, T. & Schwarz, K. Single-layer model of the hexagonal boron nitride nanomesh on the Rh(111) surface. *Phys. Rev. Lett.* **98**, 106802 (2007).

[23] Koch, H. P., Laskowski, R., Blaha, P. & Schwarz, K. Adsorption of gold atoms on the *h*-BN/Rh(111) nanomesh. *Phys. Rev. B* **84**, 245410 (2011).

[24] Koch, H. P., Laskowski, R., Blaha, P. & Schwarz, K. Adsorption of small gold clusters on the *h*-BN/Rh(111) nanomesh. *Phys. Rev. B* **86**, 155404 (2012).

[25] Kondo, J. Resistance minimum in dilute magnetic alloys. *Prog. Theor. Phys.* **32**, 37–49 (1964).

[26] Appelbaum, J. A. "$s - d$" exchange model of zero-bias tunneling anomalies. *Phys. Rev. Lett.* **17**, 91–95 (1966).

[27] Anderson, P. W. Localized magnetic states and Fermi-surface anomalies in tunneling. *Phys. Rev. Lett.* **17**, 95–97 (1966).

[28] Appelbaum, J. A. Exchange model of zero-bias tunneling anomalies. *Phys. Rev.* **154**, 633–643 (1967).

[29] Zhang, Y. *et al.* Temperature and magnetic field dependence of a Kondo system in the weak coupling regime. *Nature Comm.* **4**, 2110 (2013).

[30] Lambe, J. & Jaklevic, R. C. Molecular vibration spectra by inelastic electron tunneling. *Phys. Rev.* **165**, 821–832 (1968).

[31] Wyatt, A. F. G. & Wallis, R. H. Exchange scattering in Ti-doped Al/Al oxide/Ag tunnel junctions. i. zero magnetic field. *J. Phys. C.: Solid State Phys.* **7**, 1279–1292 (1973).

[32] Cohen-Tannoudji, C., Dupont-Roc, J. & Grynberg, G. *Atom-Photon Interaction* (Wiley and Sons, Inc., New York, 1989).
